# Bibliographical Notices

**Published:** 1844-01-27

**Authors:** 


### BIBLIOGRAPHICAL NOTICES.

*Traité clinique et pratique des Maladies des Enfans*, par MM. Barthez et Rilliet, Docteurs en Médecine, anciens internes lauréats de l'hôpital des Enfans malades de Paris, membres de la Société Médicale d'Observation, &c. &c. 3 vols. 8vo. Paris, 1843.

*Clinical and Practical Treatise on the Diseases of Children.* By Messrs. Rilliet & Barthez, &c. &c. Paris, 1843.

We had recently occasion to draw attention to the work of Dr. Condie, unquestionably, we think, the best Treatise on the Diseases of Children, which we possess in the language. Since then we have received the extensive work before us, which proceeds from that school of laborious and generally accurate observation—an emanation of recent periods—of which Louis, under the spirit of the times, may be considered as the originator, and the *Société Médicale d'Observation* as one of the fruits. Messrs. Rilliet and Barthez belong to this school, and have faithfully observed, and chronicled what they have observed in the work before us, which has been esteemed so valuable, that the *Conseil Royal* of the University of France have decided "that its use should be *authorized* in the Faculties of Medicine, and in the preparatory schools of medicine and of pharmacy of the kingdom."

The plan pursued by the authors in their description of the various diseases, is to give, as in the case of scarlatina, 1, a picture of the progress and duration of the disease; 2, an account of the prodromic phenomena; 3, a description of the eruption; 4, the concomitant symptoms of the eruption; 5, the diagnosis; 6, the state of the organs after death—the seat and nature of the disease; 7, the complications; 8, the influence of scarlatina on the diseases during the course of which it is developed; 9, the prognosis; 10, the cause; and 11, the treatment. The Essay concludes with the History of the Disease; the various epidemics; and cases;—and in the case of that on scarlatina occupies a space of upwards of 100 pages.

From this chapter we extract a part of the observations of the authors on the prophylactic treatment by belladonna.

After having enumerated the observations of many individuals—as Schenk, Massius, Hufeland, Zeuch, Gumpert, Berndt, Pitschaft, Martini, Hillenkemp, Serlo, Cohen and Kaiser, in its favor; and the adverse testimony of J. Frank, Lehmann, Teuffel, and Wagner; MM. Rilliet and Barthez conclude.

"It is difficult to discover the truth in the midst of so many contradictory opinions. It has been objected, that all children are not equally apt to contract the disease, and that if they escape the contagion after having taken belladonna, they would equally have escaped, had they not taken it. But we cannot resist the belief, when a considerable number of individuals experience immunity after the use of this prophylactic, that some virtue should be accorded to it. We think that the prophylactic treatment by belladonna may be employed without inconvenience in the doses prescribed by Hufeland, Berndt, Pitschaft and Gumpert. Moreover, in accordance with the recommendation of Dr. Kühlbrand, we may be able to prevent the disagreeable effects, which might result from the prolonged use of belladonna, by rubbing, night and morning, camphorated vinegar on the forehead and temples.

It would seem to be wise to reserve the employment of belladonna for cases in which the prevailing epidemic is very severe. If mild, but little inconvenience is sustained should children contract it, whilst they are thus protected in future. Fresh trials are necessary, and should they lead to a favorable result, they may serve also to solve several accessary questions. For example—For what length of time should the remedy be employed? What is the best mode of exhibiting it? Is the preservative effect temporary or permanent? Is it requisite to renew its employment during every epidemic? And lastly, when it has not prevented the development of the disease, does it exert any influence over its progress?"

Under the head of Paralysis, (*Paralysie essentielle*) MM. Rilliet and Barthez give the details of two cases, in one of which the disease was limited to the left upper extremity; in the other, to the lower extremities. Death occurred under the influence of a pneumonitic complication; and on dissection, no alteration of the brain or spinal marrow could be detected. Evidently, but few of these cases have fallen under the notice of the authors. The same may be said of their oxperience in Chorea, which is not a common affection in Paris. In the composition of their chapter on that subject, they state, that they have analysed nineteen cases; some of which were communicated to them by MM. Piet and Legendre.

---

*Medical Report of the Western Lying-in Hospital and Dispensary, 25, Arran-quay, for the year* 1841–42. By Fleetwood Churchill, M.D. M.R.I.A., Vice President of the Obstetrical Society, and of the Asociation of the College of Physicians; Physician of the Hospital, &c.; and R. D. Speedy, Esq. L.R.C.S.I., Surgeon to the Hospital, and Lecturer on Midwifery, &c. in the School of Medicine, Apothecaries' Hall. Dublin. 1843.

The report of the Hospital embraces a period of two years, that is, from January 1st, 1841, to December 31, 1842, inclusive. Although relief was afforded to 1506 women, owing to the irregularity with which many cases were entered in the statistical register, it was found necessary to exclude a considerable number, in order that no facts might be adduced of the accuracy of which the reporters were not certain. The records are therefore limited to the delivery of 1206 women; from these are deducted 43 cases of abortion, leaving 1163 cases of labor at full time.

"The number of children amounted to 1175, (691 males, and 484 females,) of which 63 (44 males, and 19 females,) were still-born, or died at birth; of these 12 were premature, 15 still-born, 2 putrid, 4 footling cases, 8 breech presentations, 1 head and hand presentation, 3 arm presentations, 3 funis presentations, 6 crotchet cases, 2 forceps cases, 1 placenta prævia, 4 syphilitic."

"There were *thirteen* cases of twins. In *four* cases the children presented naturally—*six* children were saved, and *two*, which were premature, died. In *six* cases one child presented the breech and the other the head—*ten* were born alive, *two* were lost. In *one* case one child presented footling and the other the head—*both* were saved. In *another*, one child presented the head and the funis, and the other the foot and unis—*both* were lost. In a *third* case both the children presented the feet and funis, and were *lost.*

In *ten* cases there was hemorrhage between the birth of the child and the expulsion of the placenta; in *six* of which manual extraction was necessary, but no unfavourable results followed.

In *six* cases flooding occurred before delivery; *three* were cases of *accidental*, and *three* of *unavoidable* hemorrhage. The rupture of the membranes was sufficient in the accidental and in one of the unavoidable cases, and the mothers and the children recovered. It was necessary to turn and deliver the child in the other two cases—*one* of the mothers died and *one* recovered: *one* of the children was saved.

*Seven* patients were attacked by *convulsions*—all recovered.

*One* fatal case of *uterine phlebitis* occurred, and several slight attacks of *hysteritis*, which were relieved by the usual treatment.

We met with *one* fatal case of *rupture of the uterus.*

*Version* was performed *six* times (1 in 243); *five* times on account of presentation of the arm—all the mothers recovered, and three children were saved, the others were putrid: and *once* because of unavoidable hemorrhage.

The *forceps* were used in *eight* cases (1 in 182). *Seven* of the mothers recovered, and the death of the remaining *one* was caused by disease of the heart.

In *eight* cases the *perforator* was employed (1 in 182). *Six* of the mothers recovered, and *two* died—one from rupture of the uterus, as recorded above, and one from disease of the liver.

Of the 1463 women attended during these two years, only *five* died, or 1 in 292. *One* sank from disease of the liver, *another* from disease of the heart, a *third* after unavoidable hemorrhage, a *fourth* from uterine phlebitis, and the *fifth* from ruptured uterus."

"Since the establishment of the hospital, seven years ago, 3211 women have been attended, of whom 15 died, or 1 in 214. There occurred

| | | | |
|---|---|---|---|
| 4 | cases of | unavoidable hemorrhage— | 1 in 802. |
| 6 | do " | accidental hemorrhage, | 1 in 525. |
| 34 | do " | hemorrhage after labor, | 1 in 94. |
| 10 | do " | convulsions (3 lost) | 1 in 321. |

Version was practised in seventeen cases—1 in 188. Sixteen recovered. Six children saved.

The forceps were used in eleven cases—1 in 291. Ten recovered. Seven children saved—two putrid.

The perforator was employed in twenty cases—1 in 160. Seventeen recovered."

The report contains many other interesting details, such as the ages of the patients, duration of labour, &c., and likewise an account of some of the more interesting cases.

For this interesting document we are indebted to our friend Dr. Churchill, of Dublin, Ireland; to whom we are also under obligations for a most valuable paper "*On Inflammation and Abscess of the Uterine Appendages,*" "read (by Dr. C.) to the Surgical Society," which we shall notice in our next number.

*Review of Dr. Caldwell's pamphlet, entitled Physiology Vindicated, in a Critique on Liebig's Animal Chemistry.* By Robert Peter, M. D., Professor of Chemistry and Pharmacy in Transylvania University. Cincinnati, 1843.

The recent work of Professor Liebig on Animal Chemistry, has created an excitement in the minds of medical philosophers rarely equalled in the present age. This has proceeded not alone from the high character of the author as a profound philosopher and most original thinker, but from the novel and startling views he has advanced in regard to the relations of Chemistry to Physiology. It was to be expected that such bold propositions when promulgated would challenge admiration and excite controversy, and such has been the case in an eminent degree. In the last number of the Examiner we extracted from an introductory of Professor Hare a few paragraphs expressive of his views of "Liebig's Physiological Speculations." The pamphlet of Professor Peter is a defence of Liebig against a most severe "Critique" by Professor Caldwell. The design of the Reviewer may be discovered from a few introductory paragraphs.

"The avowed design of the author of the pamphlet in hand, as given in his preface, is '*conservative* rather than *promotive*—to prevent the science of Physiology, in whose behalf it was conceived and resolved on, from being *injured and degraded*, rather than actually to IMPROVE and ELEVATE it;" and the belief that its publication will *not* tend to improve and elevate knowledge in general, or any branch of science in particular, but that it will rather injure and degrade; with the conviction that it is my duty to oppose, to the extent of my abilities, all such tendencies, from whatever quarter, is the strong reason which urges me to offer the following remarks to the medical public.

In undertaking, in the present instance, the disagreeable task of exposing error, many motives are presented to induce me to prefer the ease of silence. The venerable age and acknowledged standing of the author, the untiring ability with which he wields his pen, and the most ready use of argument to sustain his positions, so as often to make the 'worse *appear* the *better* reason;' the consideration that the work which he attacks in the present pamphlet, cannot be put down by a mere clash of logical arms, by the most ingenious mis-statement of its propositions, nor the strongest array of perverted or misquoted facts; the belief that any man of sense, or of clear unbiassed judgment, who had studied the productions of Liebig, would at once, without any assistance, perceive the injustice done to truth, logic, and that author, in the pamphlet of Professor Caldwell; and lastly, but not least, the fact, that, in the performance of the task I have assumed, I shall be obliged to convict the Professor, not only of practical adherence to the old mode of philosophizing, namely, that of the school of Aristotle, but also of wilful or ignorant misconstruction of facts and arguments; and what is more disagreeable, I shall be forced to expose, in his production, an amount of ignorance of science in general, and even of physiology, whose cause he undertakes to *vindicate*, and of which he has been professedly a teacher for so great a number of years, as would disgrace a tyro, and must appear incredible to the common observer."

The Review is written with ability, and although in a quiet and less dogmatical style than the "Critique," it partakes something of its temper. But, "the unkindest cut of all," to the venerable controvertist, is a review of his pamphlet contained in the last number (December, 1843) of the Western Journal of Medicine and Surgery, edited by two of his own Colleagues, and understood to be written by one of them. One or two brief extracts will show the manner in which the "Critique" is handled.

"If we could have pronounced it an able paper, though coming short of its aim; if, among its numerous arguments against animal chemistry, one solid objection had been adduced; if the author had even succeeded in pointing out the defects which, undeniably, attach to the doctrines of Liebig, we should have felt pleasure in laying before our readers all that could be urged against those popular theories by one of the most learned physiologists of our country. But after a most careful examination of what Professor Caldwell has written in this work, we cannot admit that he has set forth a single valid reason, why the chemical theories of animal heat and digestion should not be adopted. The argument evinces the learning and research which we had a right to expect, and it is proposed with an earnestness which shows that the author had a deep conviction of the soundness of his opinions, but it will surprise us if any one, who had studied the writings of Liebig, and is acquainted with the subjects in dispute, deems it so much as plausible. Whatever of force the objections urged might have had in the time of John Hunter, it is not hazardous to say, that by the great body of physiologists they are now regarded as obsolete."

The following are the concluding remarks of the reviewer:

"But we have exhausted our limits and must here bring our remarks to a close, incomplete as is our examination of the 'Critique.' That portion which remains to be noticed appears to us not less objectionable than that which has been passed in review. Truly, the reader of this singular production will have to say, as he turns over its pages,

'—— quandoque bonus dormitat Homerus!'

The learned author has slept long upon all the questions to which it relates. Nearly half a century of restless inquiry has swept unheeded by him. Its crowd of discoveries has pressed upon him in vain; his eyes have remained shut to the truths they convey. The theory with which he set out in early professional life continues to be the cherished doctrine of his advanced age, and, like another illustrious theorist in medicine, he seems to have avowed '*never to give it up till he gives up the ghost.*'"

